# Historical dataset of osteological and archaeological records for equines on Crete

**DOI:** 10.1016/j.dib.2024.110177

**Published:** 2024-02-07

**Authors:** Vera Klontza-Jaklova, Michal Smíšek, Romilda Nevěčná, Nikos Panagiotakis, Manolis Klontzas, Ricardo Fernandes

**Affiliations:** a Department of Archaeology and Museology, Faculty of Arts, Masaryk University, Arna Nováka 1, 60200 Brno, Czechia; b Institute for Natural and Cultural Heritage, Nové Sady 2, 60200 Brno, Czechia; c Free lance archaeologist, Voni, Kastelli, 70006 Herakleion, Greece; d Archaia Brno, Bezručova 15, 60200 Brno, Czechia; e Max Planck Institute of Geoanthropology, Department of Archaeology, Kahlaische Str. 10, 07745 Jena, Germany; f Department of Bioarchaeology, Faculty of Archaeology, University of Warsaw, ul. Krakowskie Przedmieście 26/28, 00-927 Warszawa, Poland; g Princeton University, Climate Change and History Research Initiative, Princeton, NJ 08544-5623, USA

**Keywords:** Cretan horse, Equine archaeology, Equine history, Equines, Horse breeding

## Abstract

We gathered evidence on the occurrence of equines in the island of Crete from the Neolithic until 1895. We relied on published archaeological and osteological records plus on historical written documents. Our dataset includes a description of the type of evidence, where this was located, and the associated absolute and relative chronologies. The collected data can be used to investigate the past spread of equids in Crete (*Equus asinus* and *Equus caballus*), their socioeconomic status, and the development of the local Cretan breed. The dataset is made available via the Abraxas data community within the Pandora data platform. This community is devoted to the historical study of horses. The dataset presented here is a component of a project tracing the history of the Cretan horse until present day.

Specifications Table

**Table:** 

Subject	*History* *Archaeology* *Zoology* *Veterinary Science and Medicine*
Specific subject area	Zooarcheology Equine history and archaeology. History of horse breeds.
Data format	Secondary (retrieved from bibliography)
Type of data	Table
Data collection	Data was compiled from archaeological and osteological articles and monographs, and from the study of historical written documents.
Data source location	Crete: Lattitude 35,50 – 34,75; Longitude 23,50 – 26,50 Santorini: Lattitude 36,50 – 36,30; Longitude 25,30 – 25,50 Repository: Pandora
Data accessibility	Repository name: Pandora Data identification number: https://www.doi.org/10.48493/tm6j-fq61 Direct URL to data: https://pandoradata.earth/dataset/vera-klontza-jaklova Instructions for accessing these data: free access
Related research article	Klontza-Jaklova, V. Panagiotakis, N. Nevěčná, R. Smíšek, M. Fernandes, R. Klontzas, M. (2023): The Cretan Horse: Still a Unique Breed? Part I: Equines on Crete from the Neolithic to the Ottoman Period, *Cheiron: The International Journal of Equine and Equestrian History*, Volume 3, Issue 2, pp.114-169. ; https://trivent-publishing.eu/img/cms/7-%20Cheiron_2-2023_V%C4%9Bra%20Klontza-Jaklov%C3%A1.pdf

## 1 Value of the Data

• The Cretan horse breed is facing extinction. Our dataset can be used to investigate the physical development of the breed, what was its socioeconomic status through time, and the uses that the breed was given by humans.
• The dataset can be used in interdisciplinary research projects combining osteology, archeology, history, military history, art history, and veterinary medicine.
• The dataset can be combined with other historical and archaeological records to offer a broad view of diachronic animal management practices in the island of Crete.

## 2 Background

The dataset is part of a comprehensive study on the specific horses bred on Crete. The primary objective is to define the Cretan horse as a specific breed. It was, therefore, necessary to collect archaeological and historical evidence of the presence and breeding of the horse on the island and, based on these sources, including osteological remains, iconography, and written sources, to assess the likelihood of continuity of breeding and, if necessary, the genetic resources of the individuals cited today. The first equids were on the island at the beginning of the Bronze Age (ca. early 3rd millennium BC). They were donkeys rather than horses [1,2]. The first specimens of Equus caballus were present in the Late Minoan Period (before 1500 BC) until the modern period [3], [4], [5], [6], [7], [8].

## 3 Data Description

Each data entry is uniquely identified via an identification field (ID_Nr). For this field integers are entered in sequence.

Each site from which equine evidence originates is uniquely identified by an integer (Site_Nr) and by a site name (Site). Site names are written in English and the most common name is given. The list of sites included in our dataset (ordered alphabetically): Afrati, Agia Triada, Akrotiri, Archaes – Fournoi, Astrakous, Avdou. Azoria, Eleutherna, Gortyn, Chalasmenos, Chamalevri, Chania, Chersonissos, Idaion Andron, Karphi, Kavousi Kastro, Kavousi Vronda, Knossos, Kommos, Lato, Lyttos, Malia, Mochlos, Monastiraki, Mouliana, Myrtos Pyrgos, Orthi Petra, Palaikastro, Phaistos, Phalasarna, Poros, Praisos, Prinias, Profitis Ilias, Sklavokambos, Syme, Tylissos, Vrokastro.

The coordinates of each site location are given in latitude (Latitude) and longitude (Longitude) fields. These are reported in decimal degrees relative to the WGS84 system. A field (Exact_loc) is used to flag if the coordinates of a location are reported by excavators (value Y) or not (value N). When not available, approximate coordinates are entered into previously mentioned fields.

The type of evidence for equines is given in a categorical field (Finds). When multiple categories refer to the same location and chronology these are entered in the same data cell and separated using semicolons. The list of evidence categories included in our dataset (ordered alphabetically): Attic pixis lid, bones, bronze bit, bronze figurine, bronze helmet, bronze mitra, bronze shield, bronze tripod, cheek piece, clay figurine, coin, faience (scarab), Fe bit, fresco, glass, horn bridle ornament, ivory seals, lamps, painted pottery, relief pithos, sarcophagus, script, seal, temple frieze, terracotta figurines, terracotta plate, weight, zoomorphic vessel.

We used binomial nomenclature to identify equine species (Species) present for a certain location and chronology. When more than one species is present for a certain location and chronology these are entered in the same data cell and separated using semicolons. An unknown category is included. The list of species included in our dataset: *Equus caballus, Equus asinus*, and Unknown.

The chronology for each equine find is given in two numeric fields expressing the minimum (Min_date) and maximum (Max_date) values for a date range. These are reported as positive CE and negative BCE values. In additional, chronological tags are used to assign each find to chronological periods/phases. Employed categories are listed in Table 1 and if a chronological assignment includes more than one period we give the earliest and latest period for the evidence separated by a dash.

**Table 1:** List of chronological periods/phases [9], [10], [11] employed to tag the chronology of each equine find. The smaller than symbol given in the field “Absolute chronology” sets the maximum value for a chronological period/phase with the minimum value given in the following row. All values are BCE up to the Roman period after which dates are CE.

Period	Phase	Abbreviation	Alternative term	Absolute Chronology
Paleolithic Period		Paleo		<10.300 BCE
Mesolithic Period		Meso		<7000
Neolithic Period		NP		<3300
Early Bronze Age	Early Minoan I	EM I	Prepalatial Period	<2700
	Early Minoan II	EM II		<2450
	Early Minoan III	EMI III		<2200
Middle Bronze Age	Middle Minoan I	MM I	Protopalatial Period	<2050
	Middle Minoan II	MM II		<1950
	Middle Minoan III	MM III	Neopalatial Period	<1750
Late Bronze Age	Late Minoan I	LM I		<1470
	Late Minoan II	LM II	Final- and Postpalatial Period	<1430
	Late Minoan III	LM III		<1150
Early Iron Age	Subminoan	SM	Subminoan	<1000
	Protogeomentric Period	PG		<900
Geometric Period		GP		<650
Orientalizing Period		OP		<600
Archaic Period		AP		<479
Classical Period		CP		<323
Hellenistic Period		HP		<66 BCE
Roman Period		RP		<324 CE
Early Byzantine Period		EByz	First Byzantine Period	<824
Arabic Period		ArabP		<961
Late Byzantine Period		LByz	Second Byzantine Period	<1204
Venetian Period		VP		<1669
Ottoman Period		Ottoman		<1895
Modern times		Modern		< present-day

The citation for each bibliographic source (Reference) is given in APA style and the associated digital object identification (DOI) is given whenever available. A notes fields (Notes) is used to provide additional information not included in aforementioned fields.

To the best of our knowledge, we have compiled all bibliographic evidence on the historical presence of equines in the island of Crete. However, our dataset does not include unpublished materials which can present a large volume of information. We do not have access to these, but we would like to encourage our colleagues to publish the material and make it available for research. We will continue to update our dataset as new data becomes available.

## 4 Experimental Design, Materials and Methods

We collected historical evidence for equines in Crete by undertaking a bibliographic research to locate and read all published archaeological and osteological articles and monographs pertaining to the topic. We also consulted historical written documents concerning the study of equines. We used the bibliography from these sources to locate additional primary sources and successively applied this approach. We also relied on contributions from expert colleagues and on online data search using the scientific search engine Google Scholar to locate primary sources. We consulted primary sources in different languages (Greek, English, Italian, German, and French) and compiled our dataset between 7000 BCE and 1895. Full references for these sources are included in our dataset together with, whenever available, a DOI.

**Fig. 1:**
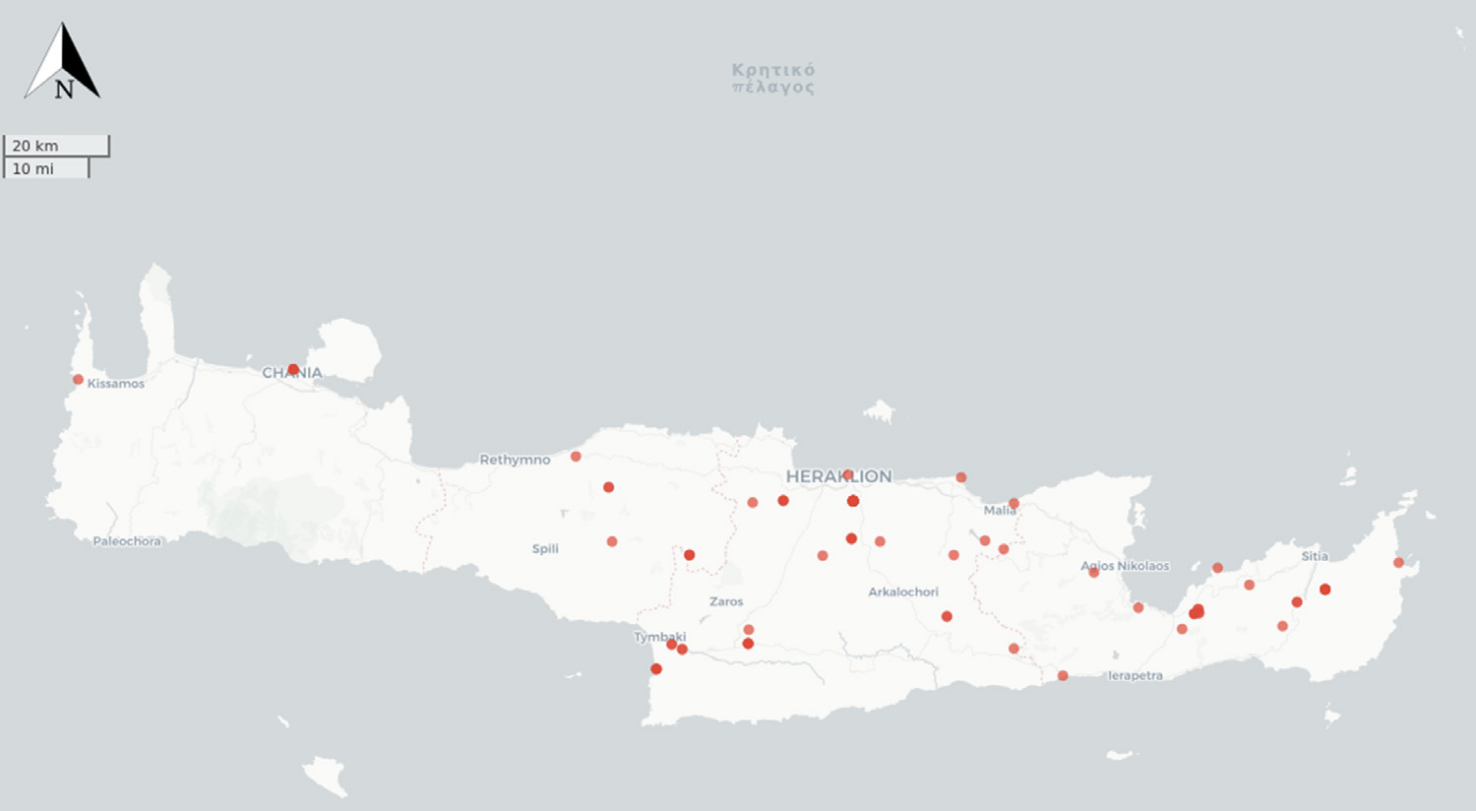
Spatial distribution of sites listed in the dataset.

In addition to the English version of the site name associated to equine evidence, we recorded the latitude and longitude coordinates for each site if this was given in original publications. Otherwise, we used the Google Maps web mapping platform to obtain an approximate location from site name. We used a field in our dataset to tag whenever an approximate location was employed. Fig. 1 shows the spatial distribution of sites listed in our dataset. Our dataset relies on some sources that include deprecated terminology to describe the typology of material finds. Whenever necessary, we report the typology of material finds using up to date archaeological terminology. We also revised original chronological assignments of finds using chronological classifications based on current consensus among the archaeological community (Table 1).

## Limitations

The completeness of the dataset in archaeological databases is affected by various factors, and this is also true for datasets containing information on horses and other equines in archaeological contexts across the island. The identification of donkeys and horses from fragmentary skeletal remains is a significant challenge. Additionally, there has been a lack of interest in certain periods of Cretan prehistory and history, resulting in incomplete information for those periods. The Bronze Age and Classical Antiquity periods have been the focus of research for a long time [9,12]. There is also a dearth of information about the Venetian and Ottoman periods, as well as much of the 20th century. Moreover, there is no stud book available that traces at least the last few generations of the island's horse population.

## Ethics Statement

All authors have read and follow the ethical requirements for publication in Data in Brief and confirm that this paper does not involve human subjects, animal experiments, or any data collected from social media platforms.

## CRediT authorship contribution statement

**Vera Klontza-Jaklova:** Conceptualization, Methodology, Validation, Data curation, Writing – original draft, Writing – review & editing. **Michal Smíšek:** Methodology, Validation, Formal analysis, Visualization, Data curation, Investigation. **Romilda Nevěčná:** Investigation. **Nikos Panagiotakis:** Investigation. **Manolis Klontzas:** Investigation. **Ricardo Fernandes:** Writing – review & editing, Formal analysis, Data curation.

## Data Availability

Historical database of equines in Crete (Original data) (https://www.pandoradata.earth/).
